# A Preliminary Study of the Suitability of Archival Bone Marrow and Peripheral Blood Smears for Diagnosis of CML Using FISH

**DOI:** 10.1155/2014/604165

**Published:** 2014-09-22

**Authors:** Alice Charwudzi, Edeghonghon E. Olayemi, Ivy Ekem, Olufunmilayo Olopade, Mariann Coyle, Amma Anima Benneh, Emmanuel Alote Allotey

**Affiliations:** ^1^Department of Chemical Pathology, University of Cape Coast School of Medical Sciences, Cape Coast, Ghana; ^2^Department of Haematology, University of Ghana Medical School, Accra, Ghana; ^3^Department of Medicine, University of Chicago, 929 East 57th Street, Chicago, IL 60637, USA; ^4^Department of Haematology, Korle-Bu Teaching Hospital, Accra, Ghana; ^5^Haematology Unit, Tamale Teaching Hospital, Tamale, Ghana

## Abstract

*Background. *FISH is a molecular cytogenetic technique enabling rapid detection of genetic abnormalities. Facilities that can run fresh/wet samples for molecular diagnosis and monitoring of neoplastic disorders are not readily available in Ghana and other neighbouring countries. This study aims to demonstrate that interphase FISH can successfully be applied to archival methanol-fixed bone marrow and peripheral blood smear slides transported to a more equipped facility for molecular diagnosis of CML.* Methods.* Interphase FISH was performed on 22 archival methanol-fixed marrow (BM) and 3 peripheral blood (PB) smear slides obtained at diagnosis. The BM smears included 20 CML and 2 CMML cases diagnosed by morphology; the 3 PB smears were from 3 of the CML patients at the time of diagnosis. Six cases had known* BCR-ABL* fusion results at diagnosis by RQ-PCR. Full blood count reports at diagnosis were also retrieved.* Result.* 19 (95%) of the CML marrow smears demonstrated the* BCR-ABL *translocation. There was a significant correlation between the* BCR-ABL* transcript detected at diagnosis by RQ-PCR and that retrospectively detected by FISH from the aged BM smears at diagnosis (*r* = 0.870; *P* = 0.035).* Conclusion.* Archival methanol-fixed marrow and peripheral blood smears can be used to detect the* BCR-ABL *transcript for CML diagnosis.

## 1. Background

Fluorescent in situ hybridization (FISH) is a sensitive molecular cytogenetic technique that enables rapid detection of chromosomal abnormalities in pathological samples for accurate diagnosis, detection of submicroscopic deletions, risk stratification, detection of minimal residual disease, and assessment of response to therapy. Unlike conventional cytogenetic techniques which require metaphase nuclei (dividing cells), FISH can be applied to nondividing interphase nuclei; hence, uncultured cells can be used [1, 2]. This makes it a useful tool in most fields for studying neoplastic disorders such as chronic myeloid leukaemia (CML). The various chromosomal abnormalities can be detected in interphase nuclei using the appropriate DNA FISH probe set [3].

A wide range of specimens such as peripheral blood and bone marrow aspirate for metaphase cell preparation; uncultured interphase cells from archival bone marrow and blood smears, paraffin-embedded tissue sections or disaggregated cells from paraffin blocks, frozen tissue, cells from lymph node aspirates or solid tumours can all be used for the analysis [3–6]. Previous studies had proved interphase FISH as a valuable and reliable contemporary approach for retrospective studies especially when fresh samples are not available, more so when additional investigative information is required to determine an underlying molecular transformation for both diagnostic and research purposes [4].

Currently, medical laboratory practice in Ghana, like other West African countries, focuses more on the diagnosis of common infectious diseases such as HIV/AIDS, malaria, and tuberculosis due to their overwhelming burden coupled with their associated social and economic challenges [7]. The underfunding of public laboratories in Ghana due to the social and economic constraints makes it difficult to develop and apply current molecular technologies for diagnosis; as a result, laboratory practitioners depend largely on morphology to diagnose malignancies using various stains. Molecular techniques such as FISH, real-time quantitative polymerase chain reaction (RQ-PCR), and flow cytometry are generally not available for routine clinical use. However, recent WHO (World Health Organization) classification of tumours especially haemopoietic and lymphoid neoplasms requires the detection of chromosomal abnormalities for accurate diagnosis and management [8].

Chronic myeloid leukaemia is a haematologic malignancy associated with a balanced reciprocal translocation between the Abelson leukaemia virus gene (*ABL*) on chromosome 9 and the break-point cluster region gene (*BCR*) on chromosome 22 forming the* BCR-ABL* fusion gene. The product of the* BCR-ABL* gene (the bcr-abl protein) is a constitutively active tyrosine kinase, which is responsible for the pathogenesis of CML [9]. Detection and monitoring of the* BCR-ABL* transcripts are essential for CML diagnosis and evaluation of patient's response to treatment with tyrosine kinase inhibitors such as Imatinib [10]. The t(9;22) chromosomal abnormality occurs in more than 95% of cases. Interphase FISH is useful in confirming the presence of the translocation [2]. In Ghana, CML accounts for 12.8% of all cases of adult leukaemia but there was no standardized method for molecular diagnosis and monitoring for minimal residual disease at the time of this study [11]. In resource-constrained countries, there is a diagnostic vacuum when it is not possible to carry out these diagnostic procedures due to lack of resources; fresh samples have to be transported to more resourced countries for analysis at great cost. This study therefore sought to determine the usefulness of transporting archival methanol fixed bone marrow and peripheral blood smears to a well-resourced country for molecular diagnosis of CML.

## 2. Methods

The study was retrospective. Ethical approval was obtained from the Ethical and Protocol Review Committee of the University of Ghana Medical School. Twenty-two (22) archival methanol-fixed bone marrow (BM) and 3 peripheral blood (PB) smear slides at diagnosis from the haematology laboratory of Korle-Bu Teaching Hospital, Ghana, were retrieved. Completely air-dried and well labelled BM and PB smears with monolayers were fixed in absolute methanol by immersion for 1 minute and allowed to air dry. Each patient's methanol-fixed smears (about 4–6 smears) were wrapped in “non-woven instrument wrap paper”, labelled with the patient's unique identification number for easy retrieval. It was then packed into a box for storage in the laboratory at room temperature. The BM smears were made up of 20 CML and 2 CMML cases diagnosed between February 2007 and January 2011; the 3 PB smears were from 3 of the CML patients at the time of diagnosis; the slides had been stored for between 10 and 48 months. The corresponding full blood count (FBC) report at diagnosis was also retrieved. Initial diagnosis for these patients was made on the basis of clinical findings and morphological examination of Leishman stained peripheral blood and bone marrow aspirate smears. Six of the CML cases had known* BCR-ABL* fusion results (using fresh peripheral blood) at diagnosis made by RQ-PCR outside Ghana. Fisher exact test was used to determine the correlation between the* BCR-ABL* transcript detected by RQ-PCR at diagnosis and that detected by FISH from the aged smears at diagnosis. The statistical significance was set at *P* ≤ 0.05. Metaphase FISH was performed on control cells; in addition, direct blood smears were prepared from the peripheral blood control for interphase FISH analysis.

### 2.1. Tissue Culture on Control Cells

Commercially prepared* BCR-ABL* fusion positive cell line in blast crisis (BV173) and a peripheral blood from a single* BCR-ABL* transcript negative volunteer were used as control. Tissue culture was performed in RPMI 1640 medium [12].

### 2.2. Pretreatment of Smears and Dropped Cells

The direct smear slides were pretreated by immersion in methanol for 1 minute, incubated in 2x standard saline citrate (2x SSC) at room temperature (RT) for 5 minutes, and digested in 0.05 mg/mL pepsin/10 mmol/HCl at 37°C for 5–10 minutes. It was then incubated in 2x SSC for 5 minutes rinsed in double distilled water and observed under a phase contrast microscope to ensure adequate lysis of the red blood cells. If red blood cells remained, an additional pepsin treatment was repeated. At this point, the desired areas for hybridization with the least amount of cell clumps were selected as previously described [4]. Glacial acetic acid: methanol fixative (1 : 3 ratios) was used to lyse red cell for direct peripheral blood control smears aged less than 3 weeks as well as two BM and one of the PB smear. Smears were then fixed in 1% formaldehyde, rinsed in 1x phosphate buffered saline (PBS), and dehydrated in 70%, 80%, and absolute ethanol [4].

### 2.3. FISH

FISH was performed using Vysis* LSI BCR/ABL* Dual Color, Dual Fusion Translocation DNA Probe (Downers Grove, IL, USA). The probe was validated by standard procedure. Hybridization and posthybridization wash conditions followed the manufacturer's protocol. Finally, the cells were counterstained with DAPI II.

Hybridized cells were examined through a Zeiss Axio fluorescence microscope using Vysis filter sets: DAPI/Spectrum Orange dual bandpass and DAPI/Spectrum Green dual bandpass, and images were captured using Axio Imager Z2 Vision 4.0 software. A total of one hundred (100) interphase nuclei were analysed per slide. The cut-off value for a positive signal was 1% [13]. Cells lacking* BCR-ABL* translocation displayed two red (R) signals, corresponding to the* ABL* probe, and two green (G) signals, corresponding to the* BCR* probe. Thus, a normal cell had 2R2G signal pattern. Cells with a balanced* BCR-ABL* translocation displayed two fusions (F) of red and green signals as well as one green and one red signal. Thus, a typical positive signal pattern for the* BCR-ABL* fusion showed 2F1R1G. The degree of positivity for* BCR-ABL* transcript was scored for each slide as percentages.

## 3. Results

### 3.1. Peripheral Blood Features at Diagnosis

The CML cases comprised of 5 females and 15 males, and the median age at diagnosis was 36 yrs (Range: 20–67 yrs). All the subjects showed marked leukocytosis in the range of 20.7–636.8 × 10^9^/L and anaemia, shown in Table 1. Anaemia, defined as haemoglobin level less than 12 g/dL [14], was observed in all the subjects. The platelet count was normal (150–450 × 10^9^/L) in 8 (40%) subjects, low (less than 150 × 10^9^/L) in two (10%) subjects, and high (greater than 450 × 10^9^/L) in 10 (50%) subjects.

### 3.2. Interphase FISH

**Figure 1:**
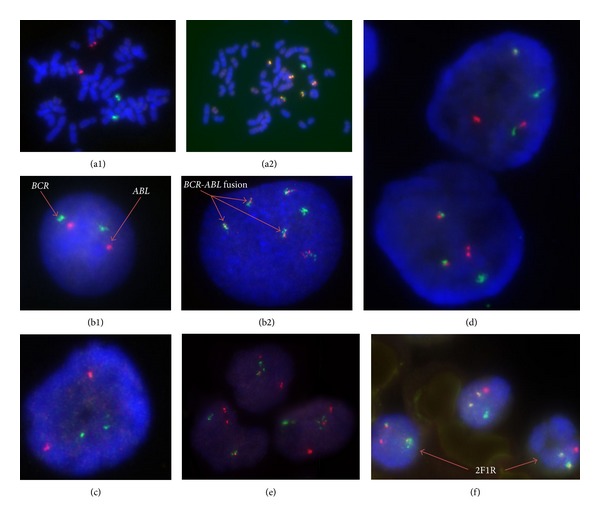
*BCR-ABL* dual colour, dual fusion translocation probe hybridized to controls, and subject's bone marrow and peripheral blood smears. ((a1) and (b1)) Two red, two green (2R2G), negative control in a metaphase cell and an interphase cell, respectively; ((a2) and (b2)) five fusion, one green (5F1G), positive control in a metaphase and an interphase cell, respectively. (c)* BCR-ABL* negative FISH signal in a subject's peripheral blood smears. (d) 2F1R1G signals in a* BCR-ABL* positive subject BM smear. (e) 1F2R1G signals in a* BCR-ABL* positive subject marrow smear. (f) 2F1R in a* BCR-ABL* positive subject's peripheral blood (red cells lysed in glacial acetic acid: methanol fixative). Negative control is from a single* BCR-ABL* negative peripheral blood donor, and BV 173 is from a* BCR-ABL* positive cell line in blastic phase. Images were taken with ×100 oil immersion objective lens.

Interphase FISH analysis was carried out in 20 CML and 2 CMML patients. Nineteen (95%) out of the 20 CML bone marrow slides at diagnosis demonstrated the *BCR-ABL* translocation. The percentage means score ± SD for the* BCR-ABL* positive smears at diagnosis was 89.5 ± 6.5 (Range: 70.0–98.0%). The two CMML subjects were *BCR-ABL* negative. The major scoring signal patterns obtained for the* BCR-ABL* positive smears were categorized into 3 groups: 1 (*n* = 17) showed the classical 2F1R1G (Figure 1(d)); group 2 (*n* = 1) showed one fusion, one red, and two green (1F1R2G) indicating* ABL* deletion; group 3 (*n* = 1) showed one fusion, two red, and one green 1F2R1G (Figure 1(e)) indicating* BCR* deletion. There was a significant correlation between the* BCR-ABL* transcript detected at diagnosis by RQ-PCR using fresh peripheral blood and that retrospectively detected by FISH from the aged bone marrow smears at diagnosis (*r* = 0.870; *P* = 0.035 by fisher exact test). Two of the PB smears showed the* BCR-ABL* translocation, and the major scoring signal was the classical 2F1R1G, with 2F1R (Figure 1(f)) as a minor scoring signal in one of the PB smears, a similar signal was seen in its corresponding BM smear. The third PB smear was negative (Figure 1(c)) (it's corresponding BM smear was also* BCR-ABL* negative).

## 4. Discussion

With the discovery of molecular markers for some neoplastic disorders, accurate assessment of the genetic characteristics of these malignancies is the hallmark for decisions on prognosis and clinical management especially with regard to targeted molecular therapies [4]. The need to identify and correlate these individual tumor features becomes a challenge when fresh pathological samples are not available or when it is not possible to carry out diagnostic procedure due to lack of resources. In resource-constrained countries, these samples may have to be transported to more resourced countries for analysis. Fresh peripheral blood samples or bone marrow aspirates that are normally relied on in the field of haematology often possess transportation challenges due to sample degradation.

Previous studies had revealed an excellent agreement in the detection and quantitation of the* BCR-ABL* translocation from results obtained from interphase cells of cultured bone marrow aspirate and peripheral blood [1]. This study was performed to determine the usefulness of interphase FISH as a reliable, reproducible, and quantitative molecular diagnostic technique on methanol fixed archival bone marrow and peripheral blood smears stored at room temperature which can be transported to a better equipped laboratory without fear of sample degradation.

The data obtained shows that the majority (89%) of the subjects showed the classical* BCR-ABL* dual fusion (2F1R1G) pattern at diagnosis. Approximately 11% demonstrated the unusual signal patterns 1F1R2G suggesting* ABL* deletion and 1F2R1G suggesting* BCR* deletion. Such deletions have been reported in 10–15% of patients and may confer a poor prognosis in CML patients in the advanced phase [15, 16]. There was a significant correlation between the* BCR-ABL* transcripts levels detected at diagnosis by RQ-PCR in six CML patients using fresh peripheral blood samples prior to commencement of treatment and their corresponding archival bone marrow smears at diagnosis using interphase FISH. This implies that aged methanol fixed bone marrow smear specimens are as reliable as fresh blood specimens, for quantification of the leukaemia burden at diagnosis. Hence, methanol fixed bone marrow and peripheral blood smears can conveniently be used for the diagnosis of CML using FISH technique. This study demonstrated that* BCR-ABL* transcript could be detected by FISH using aged bone marrow and peripheral blood smears.

Interphase FISH technique was applied directly without tissue culture to methanol fixed bone marrow and peripheral blood smears (stored at room temperature) retrieved from the haematology laboratory. This makes this technique rapid, simple, and less expensive since culture and other logistics will not be required.

However, thin films (smears) with monolayer sections treated with glacial acetic acid/methanol fixative for red cell lysis gave a better result, and although some red cells are not lysed, all the white cells remain intact. Also, the pepsin digestion step has to be monitored since overdigestion can lead to loss of all the cells on the slide.

## 5. Conclusions

The result confirms that archival bone marrow and peripheral blood smears can successfully be used to detect and quantify the* BCR-ABL* transcript to diagnose CML. The* BCR-ABL* fusion was reliably detected. This has significant implications for the setting-up of regional diagnostic centres of excellence in the main referral hospital, in Ghana as well as in the African continent. Since it may not be feasible in the near future to establish well-equipped medical laboratories in all regional hospitals, samples from the other regions in Ghana and even neighbouring countries can be collected, fixed, and sent to these centres, where molecular diagnosis will be made and the results relayed promptly via the internet. This will greatly improve the diagnosis, treatment, and monitoring of response to treatment of many malignant diseases on the continent. We recommend carrying further studies with a larger sample size to determine how long methanol fixed marrow and peripheral smears can be stored at room temperature before degradation set in.

## Figures and Tables

**Table 1:** Pretreatment full blood count profile of study population.

Parameter	Study subject, *N* = 20	Ranges
	Median	
Leucocyte count (×10^9^/L)	253.1	20.7–636.8
Platelet count (×10^9^/L)	446.2	46.0–1051.0
Haemoglobin (g/dL)	8.2	5.2–11.5
Neutrophils (%)∗∗	67.6	45.7–89.3
Eosinophils (%)∗∗	2.2	0.1–12.3
Basophils (%)∗∗	4.5	0.0–25.0

Table 1 shows the full blood count profile of the 20 subjects at diagnosis. NB: % = percentage. ∗∗Absolute values for the differential counts were not available for majority of subjects at diagnosis; hence, percentage differential count was used.

## Abbreviations

CML: Chronic myeloid leukaemia
CMML: Chronic myelomonocytic leukaemia
FISH: Fluorescent in situ hybridization
RQ-PCR: Real-time quantitative polymerase chain reaction
BCR: Break-point cluster region gene
ABL: Abelson leukaemia virus gene.
